# The IRE1α-XBP1 Signaling Axis Promotes Glycolytic Reprogramming in Response to Inflammatory Stimuli

**DOI:** 10.1128/mbio.03068-22

**Published:** 2022-12-08

**Authors:** Bevin C. English, Hannah P. Savage, Scott P. Mahan, Vladimir E. Diaz-Ochoa, Briana M. Young, Basel H. Abuaita, Gautam Sule, Jason S. Knight, Mary X. O’Riordan, Andreas J. Bäumler, Renée M. Tsolis

**Affiliations:** a Department of Medical Microbiology and Immunology, University of California—Davis, Davis, California, USA; b Department of Microbiology and Immunology, University of Michigan, Ann Arbor, Michigan, USA; c Division of Rheumatology, Department of Internal Medicine, University of Michigan, Ann Arbor, Michigan, USA; Yale University School of Medicine

**Keywords:** *Brucella*, endoplasmic reticulum, immunometabolism, innate immunity

## Abstract

Immune cells must be able to adjust their metabolic programs to effectively carry out their effector functions. Here, we show that the endoplasmic reticulum (ER) stress sensor Inositol-requiring enzyme 1 alpha (IRE1α) and its downstream transcription factor X box binding protein 1 (XBP1) enhance the upregulation of glycolysis in classically activated macrophages (CAMs). The IRE1α-XBP1 signaling axis supports this glycolytic switch in macrophages when activated by lipopolysaccharide (LPS) stimulation or infection with the intracellular bacterial pathogen Brucella abortus. Importantly, these different inflammatory stimuli have distinct mechanisms of IRE1α activation; while Toll-like receptor 4 (TLR4) supports glycolysis under both conditions, TLR4 is required for activation of IRE1α in response to LPS treatment but not B. abortus infection. Though IRE1α and XBP1 are necessary for maximal induction of glycolysis in CAMs, activation of this pathway is not sufficient to increase the glycolytic rate of macrophages, indicating that the cellular context in which this pathway is activated ultimately dictates the cell’s metabolic response and that IRE1α activation may be a way to fine-tune metabolic reprogramming.

## INTRODUCTION

It is becoming increasingly evident that the metabolism of immune cells is closely tied to their effector functions; thus, immune cells must be able to alter their metabolic programs in response to different stimuli. Macrophages have different activation states and associated metabolic programs that enable them to carry out different physiological roles. While there are likely many different activation profiles *in vivo*, one activation state that has been studied extensively are classically activated macrophages (CAMs). These CAMs, sometimes referred to as M1 macrophages, have a glycolysis-driven metabolism, allowing for the rapid production of ATP and antimicrobial products, such as reactive oxygen and nitrogen species (1). A variety of stimuli can induce CAMs, including certain cytokines and bacterial pathogens or products, such as lipopolysaccharide (LPS) (1) and the intracellular pathogen Brucella abortus (2–4).

The endoplasmic reticulum (ER) is an organelle that plays a key role in maintaining cellular homeostasis. When ER function is perturbed, the cell experiences ER stress and initiates the unfolded protein response (UPR), a collection of linked signaling cascades, to overcome the initiating stress and return to homeostasis. The most evolutionarily conserved branch is that of the ER stress sensor IRE1α. Upon activation, IRE1α oligomerizes and transautophosphorylates, activating its RNase activity (5). One key function of activated IRE1α is the excision of a noncanonical intron from the unspliced XBP1 transcript (*XBP1u*), resulting in the spliced XBP1 transcript (*XBP1s*), which encodes a transcription factor that regulates a wide range of genes involved in a variety of cellular processes (6, 7).

UPR signaling is closely linked to the immune system. IRE1α signaling leads to activation of JNK (8), NF-κB (9, 10), and NOD1 and NOD2 (11, 12), while XBP1 directly regulates the expression of proinflammatory cytokines (13, 14). The UPR is activated in many different immune cells after stimulation, including T cells (15), natural killer (NK) cells (16), and macrophages (17–19). Many intracellular pathogens induce ER stress in their host cells (20, 21), including Brucella spp., which use their type IV secretion systems (T4SS) to interact extensively with the ER (22), ultimately leading to UPR activation (23–27). IRE1α has been shown to be phosphorylated upon Brucella infection (23, 28) and to form puncta throughout infected cells (23). Though it is well established that IRE1α plays an important role in the development and effector functions of immune cells, the links between IRE1α activation and innate immunity remain poorly understood. Thus, we set out to determine how IRE1α influences the activation of macrophages in response to inflammatory stimuli.

## RESULTS

### IRE1α supports lactate production and CAM gene expression during *B. abortus* infection or LPS stimulation in macrophages.

**FIG 1:**
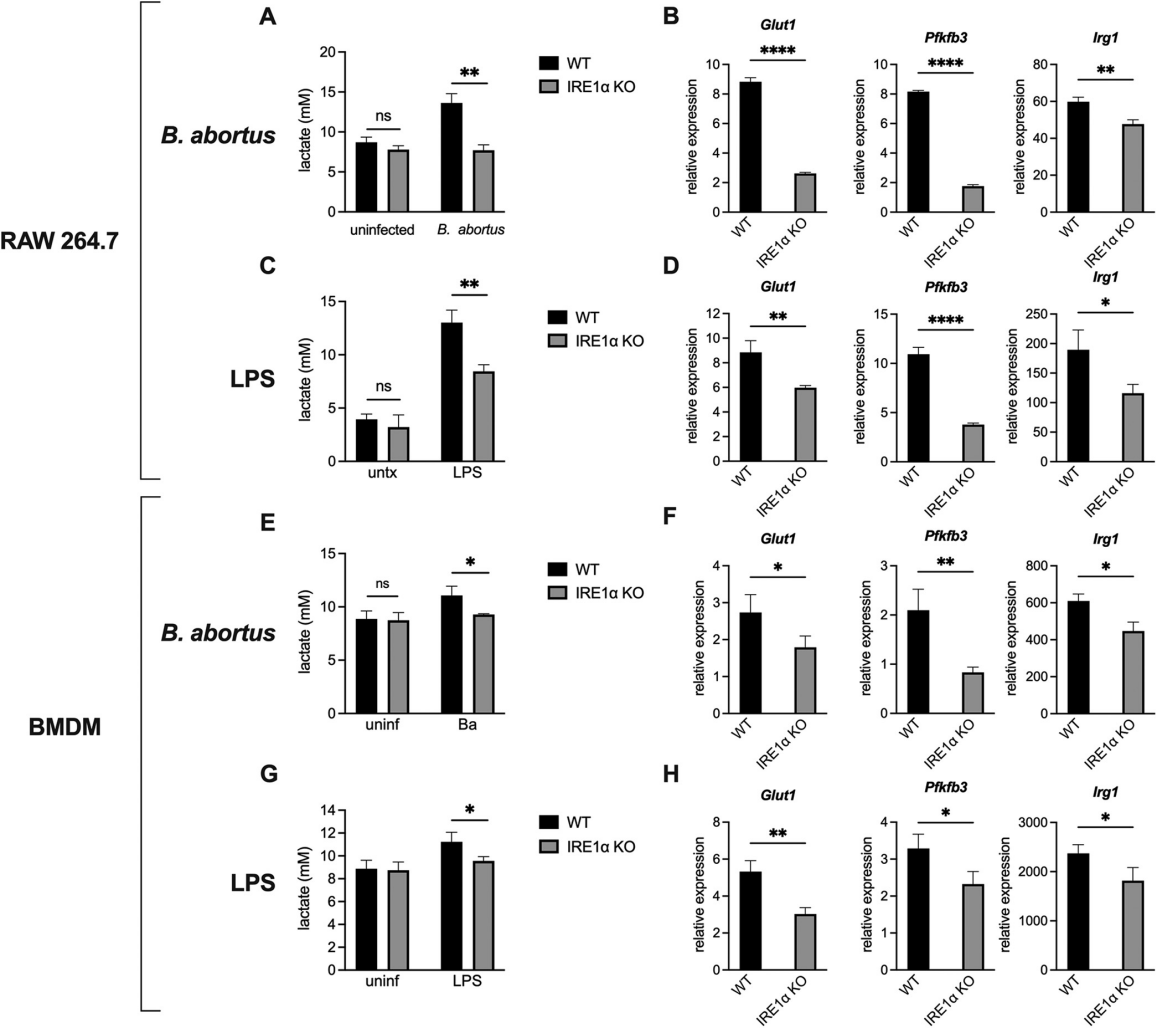
IRE1α supports lactate production and glycolytic gene expression during B. abortus infection or LPS stimulation in macrophages. Wild-type (WT) and IRE1α knockout (KO) RAW 264.7 cells were infected with B. abortus (Ba) for 48 h (A and B) or stimulated with 100 ng/mL Salmonella LPS for 24 h (C and D). Supernatant lactate was quantified (A and C), and the relative expression of the indicated genes normalized to uninfected controls was assessed by reverse transcription-quantitative PCR (RT-qPCR) (B and D). (E to H) Same as panels A to D, but with WT (LysM-Cre^−^ *Ern1^fl/fL^*) or IRE1α KO (LysM-Cre^+^ *Ern1^fl/fL^*) BMDMs. The data are presented as means of triplicate wells ± the standard deviations (SD). *, *P ≤ *0.05; **, *P ≤ *0.01; ***, *P ≤ *0.001; ****, *P ≤ *0.0001; ns, no statistical difference (Student two-tailed *t* test).

During *in vitro* infection with B. abortus, macrophages shift their metabolism to be more glycolysis-driven (2–4). Consistent with this, we observed that RAW 264.7 macrophage-like cells acidify the culture media during infection, as indicated by the yellowing of the pH indicator phenol red in the media. However, we noticed that the media on IRE1α knockout (KO) RAW 264.7 cells (29) was not yellowing to the same extent as the media on wild-type (WT) cells during infection; thus, we hypothesized that the IRE1α-deficient cells were producing less lactate. Indeed, the IRE1α KO RAW 264.7 cells produce less lactate after B. abortus infection compared to WT cells (Fig. 1A). We also assessed the expression of two genes involved in glycolysis, *Glut1*, which encodes a glucose importer, and *Pfkfb3*, which encodes a glycolytic enzyme, as well as *Irg1* (also called *Acod1*), which is a marker of CAMs (30). Similar to what we observed with lactate levels, the IRE1α KO RAW cells show an impaired induction of these genes (Fig. 1B), suggesting that IRE1α supports the Brucella-induced glycolytic switch in macrophages.

It has been reported previously that IRE1α contributes to the intracellular replication of Brucella (23, 25, 26, 31–33), and we also observed that IRE1α-deficient macrophages had a slight reduction in bacterial burden during infection (see Fig. S1A and B in the supplemental material). Thus, to ensure that the reduced glycolytic shift in IRE1α KO macrophages was not secondary to reduced bacterial burden, we tested an additional stimulus. LPS is commonly used to polarize CAMs and activates IRE1α through Toll-like receptor 4 (TLR4) signaling (13, 34). When treated with LPS from Salmonella enterica serotype Typhimurium, a potent TLR4 agonist, IRE1α KO RAW 264.7 cells had a reduced glycolytic response (Fig. 1C and D), consistent with what we observed with B. abortus infection.

10.1128/mbio.03068-22.1 FIG S1 IRE1α supports B. abortus intracellular replication. WT or IRE1α KO RAW 264.7 cells (A) and WT (LysM-Cre^−^ *Ern1^fl/fL^*) or IRE1α KO (LysM-Cre^+^ *Ern1^fl/fL^*) BMDMs (B) were infected with B. abortus and intracellular bacterial burden was enumerated by determining the colony-forming units (CFU) at the indicated time points. Data points are means of triplicate wells ± the SD. *, *P ≤ *0.05; **, *P ≤ *0.01; ***, *P ≤ *0.001 (Student two-tailed *t* test). Download FIG S1, TIF file, 0.1 MB. Copyright © 2022 English et al. 2022 English et al. https://creativecommons.org/licenses/by/4.0/ This content is distributed under the terms of the Creative Commons Attribution 4.0 International license.

Because RAW 264.7 cells are a murine cancer-derived cell line, we wanted to confirm our findings in primary cells. To this end, we tested bone marrow-derived macrophages (BMDMs) from IRE1α conditional knockout animals (*LysM-Cre^+/−^ Ern1a^fl/fL^*) and their WT littermate controls (*LysM-Cre^−/−^ Ern1a^fl/fL^* [35]). Consistent with our observations with RAW 264.7 cells, the IRE1α-deficient BMDMs also showed reduced lactate production and glycolytic gene expression after B. abortus infection or LPS treatment (Fig. 1E to H). Together, these data demonstrate that IRE1α supports macrophage glycolytic reprogramming in response to inflammatory stimuli.

### XBP1 supports lactate production and CAM gene expression during *B. abortus* infection or LPS stimulation in macrophages.

**FIG 2:**
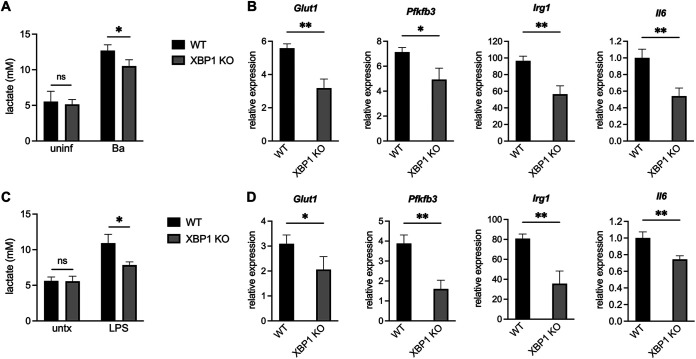
XBP1 promotes lactate production and the expression of glycolytic and inflammatory genes during B. abortus infection or LPS treatment. WT and XBP1 KO RAW 264.7 cells were infected with B. abortus (Ba) for 48 h (A and B) or treated with 100 ng/mL Salmonella LPS for 24 h (C and D). Supernatant lactate was quantified (A and C) and relative expression of the indicated genes was assessed by RT-qPCR (B and D). Expression levels of *Glut1*, *Pfkfb3*, and *Irg1* were normalized to uninfected controls. Because it is not detected in unstimulated cells, *IL-6* expression was normalized to the infected or LPS-stimulated WT cells. The data are presented as means of triplicate wells ± the SD. *, *P ≤ *0.05; **, *P ≤ *0.01; ns, no statistical difference (Student two-tailed *t* test).

We then wanted to determine how IRE1α was promoting glycolysis in CAMs. IRE1α is both a kinase and RNase, and we wondered which of these enzymatic functions was influencing macrophage metabolism. Treatment of macrophages with 4μ8c, which inhibits the RNase activity of IRE1α without affecting its kinase activity (36), led to reduced expression of glycolytic genes after B. abortus infection or LPS stimulation (see Fig. S2). There are two major outcomes of IRE1α endonuclease activity: splicing of the unspliced XBP1 mRNA (*XBP1u*), forming the spliced XBP1 transcript (*XBP1s*), which encodes a transcription factor, and regulated IRE1α-dependent decay (RIDD), a process where specific RNA species are degraded (21). Because XBP1 regulates different metabolic states in a variety of cells (15, 16, 18, 19, 37), we chose to focus on XBP1. We used CRISPR/Cas9 to generate XBP1 KO RAW 264.7 cells (see Fig. S3). These cells had reduced expression of *Il6*, a direct XBP1s target (13), after Brucella infection or LPS stimulation, further demonstrating that this pathway is activated under these inflammatory conditions (Fig. 2B and D). Like IRE1α KO macrophages, these XBP1 KO macrophages also had a reduced glycolytic response to B. abortus infection (Fig. 2A and B) or LPS stimulation (Fig. 2C and D). Thus, the IRE1α-XBP1 signaling axis promotes the glycolytic switch in macrophages in response to inflammatory stimuli.

10.1128/mbio.03068-22.2 FIG S2 The RNase activity of IRE1α supports the glycolytic response to B. abortus infection or LPS stimulation in macrophages. BMDMs were infected with B. abortus for 48 h (A) or stimulated with Salmonella LPS for 24 h (B) and concurrently treated with 50 μM 4μ8c. Relative expression of the indicated genes normalized to uninfected or unstimulated controls was assessed by RT-qPCR. The data are presented as means of triplicate wells ± the SD. *, *P ≤ *0.05; **, *P ≤ *0.01 (Student two-tailed *t* test). Download FIG S2, TIF file, 0.3 MB. Copyright © 2022 English et al. 2022 English et al. https://creativecommons.org/licenses/by/4.0/ This content is distributed under the terms of the Creative Commons Attribution 4.0 International license.

10.1128/mbio.03068-22.3 FIG S3 Generation of XBP1 KO cells. (A) Electropherograms from WT and XBP1 KO RAW 264.7 cells showing the nonsense mutation introduced by CRISPR/Cas9. (B and C) WT and KO cells were treated with 500 nM thapsigargin (Tg) for 18 h. XBP1s protein levels were assessed by Western blotting. (C) The relative expression of the XBP1s target *ERdj4* normalized to untreated controls was assessed in Tg-treated cells by RT-qPCR. The data are presented as means of triplicate wells ± the SD. *, *P ≤ *0.05; **, *P ≤ *0.01 (Student two-tailed *t* test). Download FIG S3, TIF file, 1.0 MB. Copyright © 2022 English et al. 2022 English et al. https://creativecommons.org/licenses/by/4.0/ This content is distributed under the terms of the Creative Commons Attribution 4.0 International license.

### Glucose import of infected macrophages correlates with bacterial burden and is reduced in IRE1α or XBP1 KO macrophages.

**FIG 3:**
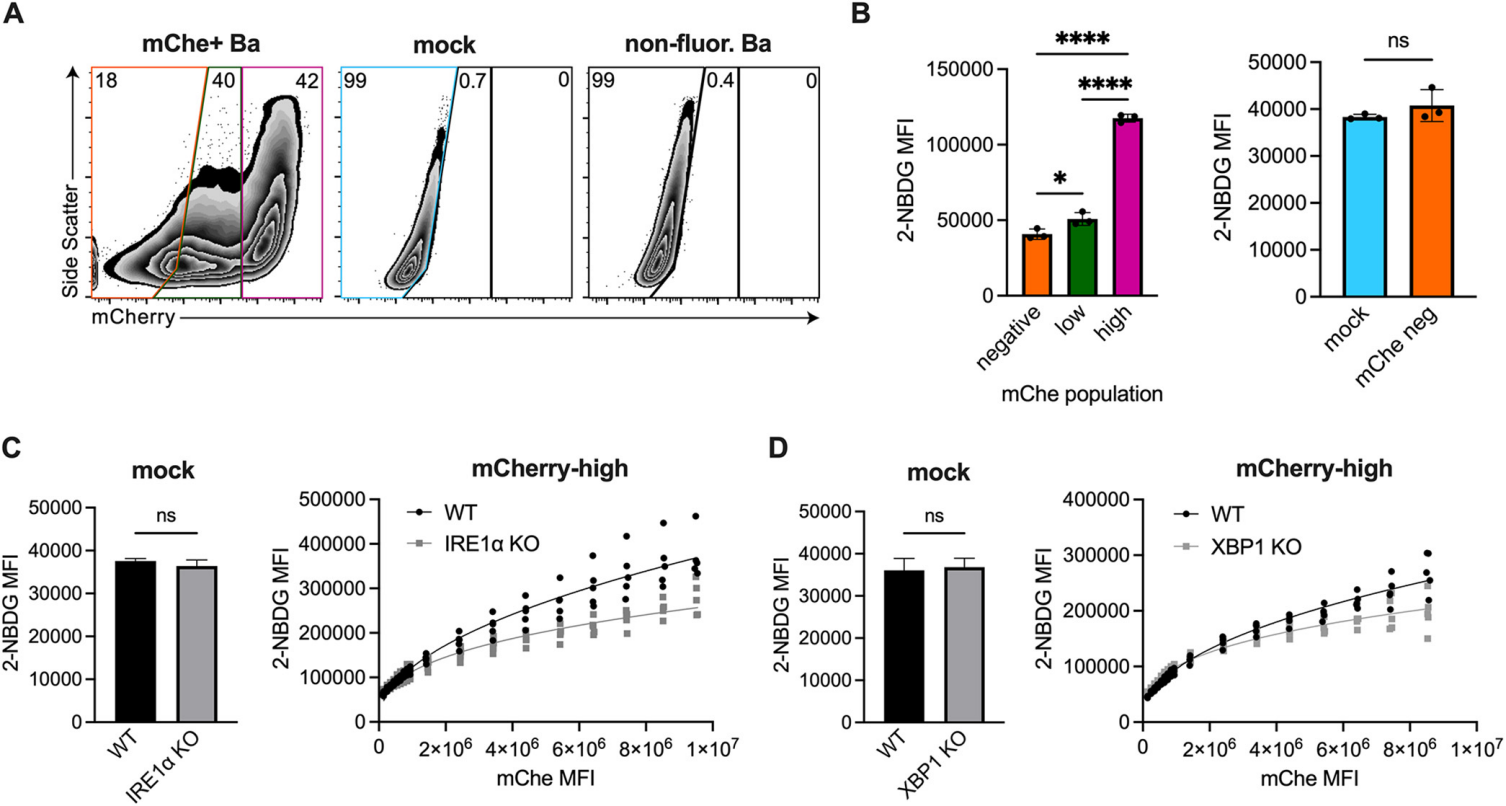
Glucose import of infected macrophages correlates with bacterial burden and is reduced in IRE1α or XBP1 KO macrophages. (A and B) WT RAW 264.7 cells were mock infected or infected with mCherry (mChe)-expressing B. abortus for 48 h and then stained with fluorescent glucose analog 2-NBDG. Cells were then gated based on mCherry signal. (A) Representative fluorescence-activated cell sorting plots showing mock or infected RAW 264.7 cells, gated on all live cells. RAW 264.7 cells infected with a wild-type non-mCherry-expressing strain is shown as an mCherry-negative control. (B) The MFI of 2-NBDG was calculated within the indicated populations. (C and D) RAW 264.7 cells of the indicated genotypes were mock infected or infected with mCherry-expressing B. abortus for 48 h before 2-NBDG staining. (Left) 2-NBDG MFIs of mock-infected cells. (Right) 2-NBDG MFI for the mCherry-high populations after binning based on mCherry signal. Dots represent individual wells, columns are means, and error bars are the SD. *, *P ≤ *0.05; **, *P ≤ *0.01; ***, *P ≤ *0.001; ****, *P ≤ *0.0001; ns, no statistical difference (as determined by a Student two-tailed *t* test, except for the left side of panel B) which was analyzed using one-way analysis of variance [ANOVA] with Tukey’s *post hoc* test.

While infection leads to increased lactate and expression of glycolytic genes (Fig. 1 and 2), the magnitude of this increase was small in some cases, leading us to hypothesize that uninfected cells in our bulk assays, such as reverse transcription-quantitative PCR (RT-qPCR) and lactate measurements, may be masking the specific effect of B. abortus on the metabolic state of infected cells. To look at glycolysis on a single-cell level, we used 2-[*N*-(7-nitrobenz-2-oxa-1,3-diazol-4-yl) amino]-2-deoxy-d-glucose (2-NBDG), an unmetabolizable fluorescent glucose analog that accumulates inside cells proportionately to their glucose import rate and thus can be used to assess their glycolytic rate (38). To assess the bacterial burden of individual cells, we used a WT B. abortus strain that expresses mCherry (4). We observed that the glucose import rate of cells correlated with bacterial burden (Fig. 3A and B), suggesting that increased glycolysis is a cell-intrinsic effect of infection. Indeed, the glucose import of mock-infected cells was equivalent to that of uninfected bystander cells (Fig. 3B). These data suggest that B. abortus acts directly on infected cells to promote glycolysis and that increased glycolysis is not simply a result of paracrine signaling from infected cells.

We hypothesized that IRE1α-XBP1 signaling contributed to glucose import during B. abortus infection, as this signaling pathway was necessary for maximal expression of the glucose importer *Glut1* (Fig. 1 and 2). Consistent with our previous data, mock-infected IRE1α and XBP1 KO macrophages had comparable glucose import compared to wild-type cells (Fig. 3C and D). When assessing the glucose import of highly infected cells, we wanted to ensure we were comparing cells with comparable bacterial burdens. Because IRE1α supports the replication of B. abortus (23, 25, 26, 31–33) (see Fig. S1), we were concerned that any observed reduction in glucose import by the IRE1α knockout macrophages could be due to reduced bacterial burden. To overcome this limitation, we binned the data across the range of mCherry signal, resulting in comparable mean fluorescence intensities (MFIs) and thus comparable bacterial burdens within each bin. We then compared the 2-NBDG signal within each bin. Across the bins, the glucose import rate of WT macrophages was higher than that of the IRE1α and the XBP1 KO macrophages (Fig. 3C and D). Together, these data provide more evidence that IRE1α-XBP1 signaling promotes glycolysis during B. abortus infection.

### IRE1α and XBP1 are required for maximal glycolytic flux after LPS stimulation.

**FIG 4:**
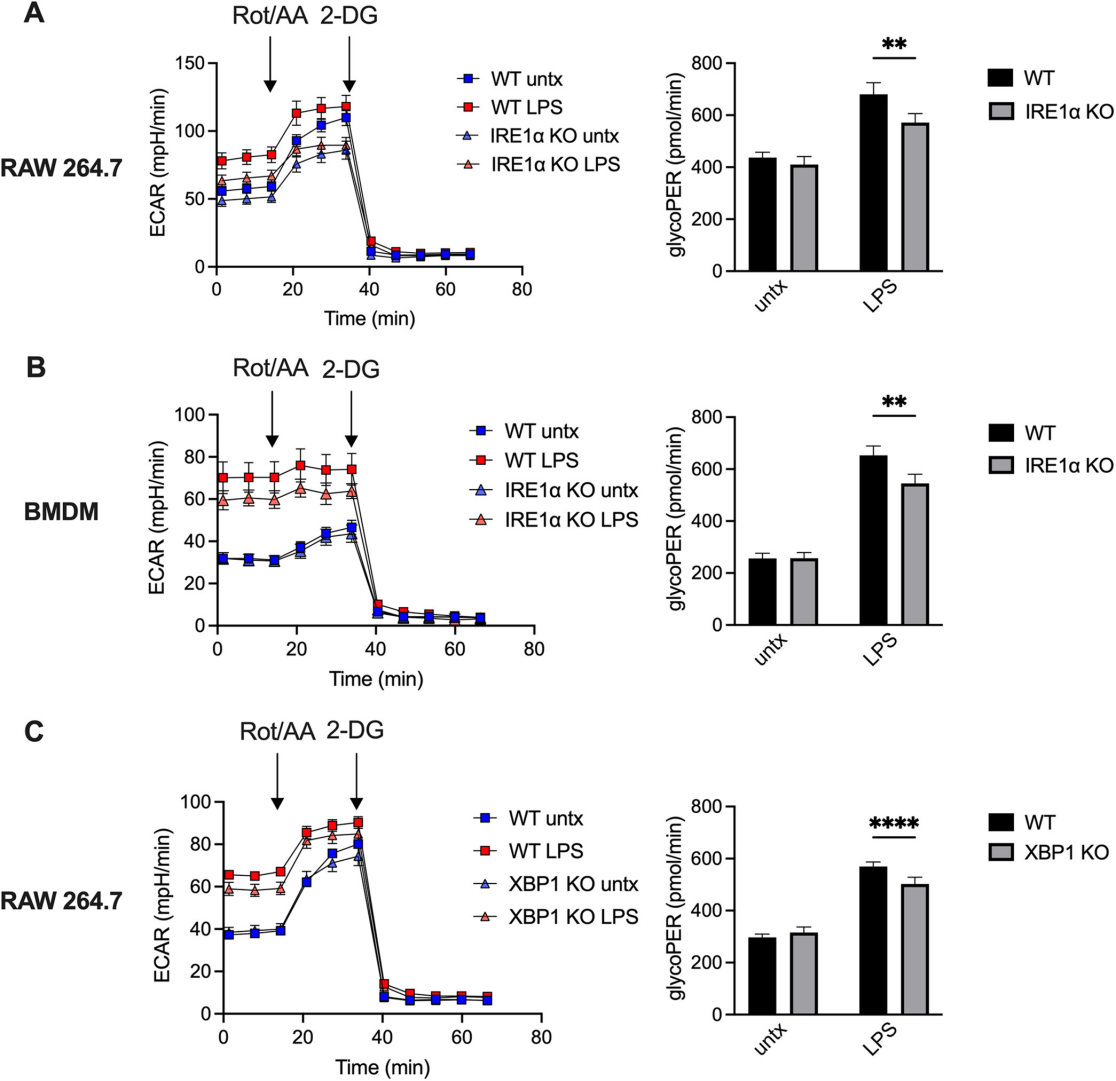
IRE1α and XBP1 support glycolysis after LPS stimulation. (A to C) WT or IRE1α KO RAW 264.7 cells (A), WT (LysM-Cre^−^ *Ern1^fl/fL^*) or IRE1α KO (LysM-Cre^+^ *Ern1*^fl/fL^) BMDMs (B), and WT or XBP1 KO RAW 264.7 cells (C) were stimulated with 100 ng/mL Salmonella LPS for 6 h before the assessing extracellular acidification rate (ECAR), with rotenone/antimycin A (Rot/AA) and 2-doxyglucose (2-DG) treatments, as indicated (left). The proton efflux rate from glycolysis (glycoPER) was calculated as a more specific assessment of glycolytic flux (right).

Though we demonstrated that IRE1α and XBP1 contribute to lactate accumulation, glycolytic gene expression, and glucose import after macrophage stimulation, these are indirect measurements of glycolysis, and we wanted to directly measure glycolytic flux of stimulated macrophages in real time. To this end, we assessed the extracellular acidification rate (ECAR) of our IRE1α- and XBP1-deficient macrophages after LPS stimulation. However, factors other than glycolytic rate, such as mitochondrial production of CO_2_, can contribute to ECAR; thus, we also assessed the proton efflux rate from glycolysis (glycoPER) specifically. Consistent with our previous data, the KO macrophages showed reduced ECAR and glycoPER after LPS stimulation (Fig. 4), further demonstrating that the IRE1α-XBP1 signaling axis promotes glycolytic flux.

### TLR4 supports glycolysis in macrophages but is not required for IRE1α activation during *B. abortus* infection.

**FIG 5:**
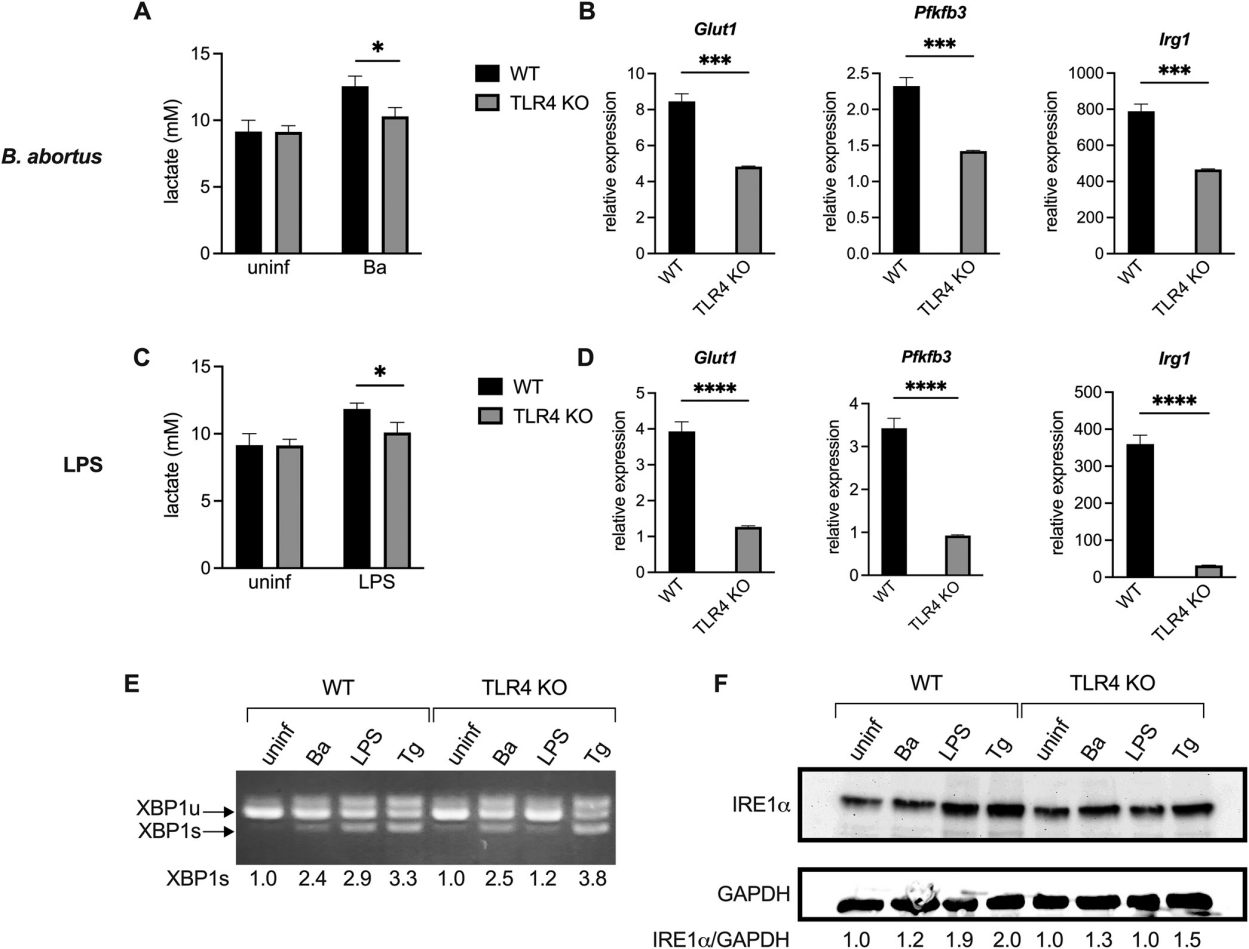
TLR4 supports glycolysis but not activation of IRE1α during B. abortus infection. BMDMs from WT or TLR4 KO mice were infected with B. abortus (Ba) for 48 h, treated with 100 ng/mL Salmonella LPS for 24 h, or treated with 250 nM thapsigargin (Tg) for 24 h. (A and C) Supernatant lactate was quantified. (B and D) Relative expression of the indicated genes normalized to uninfected controls was assessed by RT-qPCR. (E) *XBP1* splicing was assessed via nonquantitative RT-PCR. The densitometry of the XBP1s band relative to uninfected samples for each genotype is reported below. (F) IRE1α protein levels were assessed by Western blotting. Densitometry of the IRE1α band normalized to the GAPDH band and relative to uninfected for each genotype is reported below. Lactate and expression data are presented as means of triplicate wells ± the SD. *, *P ≤ *0.05; **, *P ≤ *0.01; ***, *P ≤ *0.001; ****, *P ≤ *0.0001 (Student two-tailed *t* test).

We then wondered if both Salmonella LPS and B. abortus were activating the IRE1α-XBP1 signaling pathway in the same manner. LPS activates IRE1α via TLR4 (13, 34), and Salmonella LPS is a strong TLR4 agonist. Though Brucella spp. have a modified LPS that is a weak TLR4 agonist (39, 40) and encode an effector that downregulates TLR4 signaling during infection (41, 42), TLR4 has been shown to play a role in the response to Brucella infection (43, 44). Thus, we generated BMDMs from WT and TLR4 KO mice. As expected, TLR4 KO BMDMs show a severely attenuated glycolytic response to LPS stimulation (Fig. 5C and D). After B. abortus infection, TLR4 KO BMDMs also show a decreased upregulation of glycolysis (Fig. 5A and B), which is intriguing since B. abortus reduces activation of TLR4 by its LPS during infection (45, 46).

While these data demonstrate that TLR4 supports the induction of glycolysis in classically activated macrophages, we next wanted to determine whether TLR4 was activating the IRE1α-XBP1 signaling axis or acting in a parallel pathway. We examined IRE1α activation by assessing *XBP1* splicing and IRE1α levels, since IRE1α activation leads to IRE1α upregulation in a positive-feedback loop (47). As expected, LPS treatment of TLR4 KO macrophages failed to induce significant *XBP1* splicing or IRE1α upregulation. However, TLR4 KO macrophages showed robust *XBP1* splicing and modest IRE1α upregulation during B. abortus infection (Fig. 5E and F), demonstrating that TLR4 is not required for IRE1α activation during B. abortus infection.

### Maximal glucose import by macrophages is dependent on the type IV secretion system during *B. abortus* infection.

Because TLR4 is not required for the IRE1α-mediated induction of glycolysis during B. abortus infection, we then interrogated how B. abortus was promoting glycolysis in macrophages. B. abortus uses its type IV secretion system (T4SS) to interact extensively with the host cell ER, resulting in robust intracellular replication and the induction of ER stress and subsequent IRE1α activation (22). Thus, we investigated the role of the T4SS in Brucella-induced glycolytic induction in macrophages. We expressed mCherry in our *virB2* mutant bacteria, which lack the T4SS, and in the complemented strain (48). Because the T4SS is required for intracellular replication, we increased the multiplicity of infection (MOI) used for the T4SS mutant, since we have previously observed that this increased MOI results in macrophages containing a high burden of the T4SS mutant (49).

**FIG 6:**
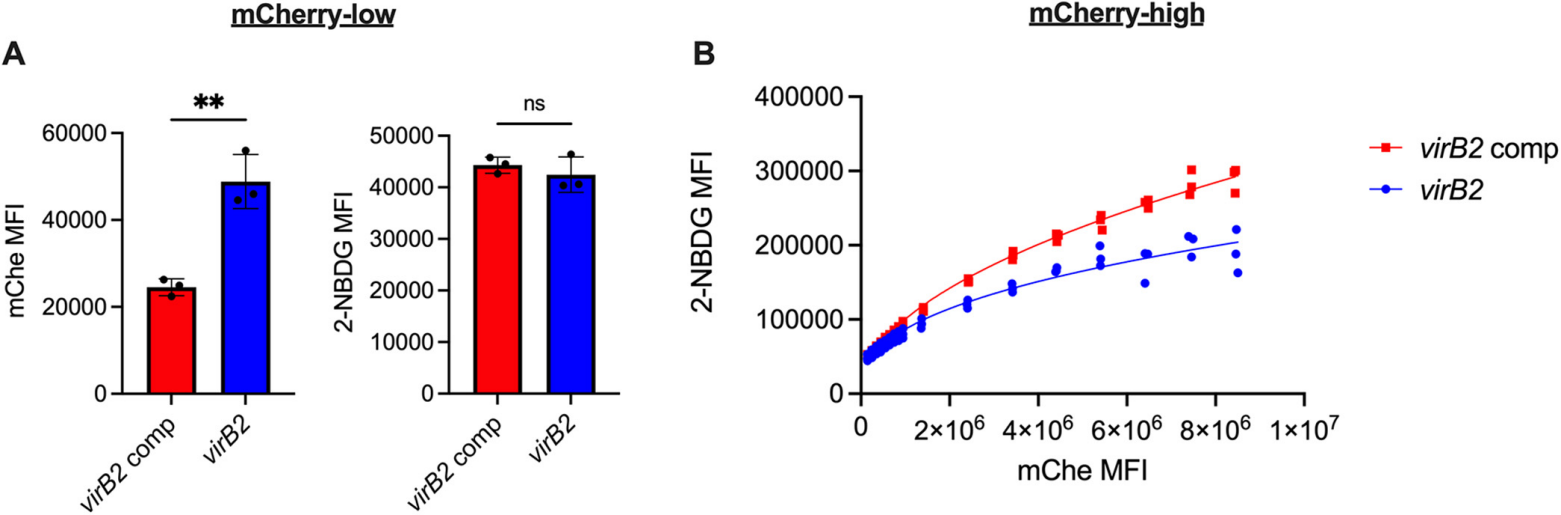
The type IV secretion system promotes glucose import by macrophages during B. abortus infection. RAW 264.7 cells were infected with the T4SS-deficient mCherry-expressing *virB2* mutant at an MOI of 2,000 or the mCherry-expressing complemented *virB2* strain at an MOI of 100 and then stained with 2-NBDG after 48 h. (A) MFIs of mCherry and 2-NBDG for the mCherry-low population of RAW 264.7 cells infected with the indicated B. abortus strains. (B) 2-NBDG MFI for the mCherry-high populations infected with the indicated B. abortus strains after binning based on mCherry signal. Dots represent individual wells, columns are means, and error bars are the SD. **, *P ≤ *0.01; ns, no statistical difference (Student two-tailed *t* test).

Even though all strains express the same level of fluorescence (see Fig. S4A), the mCherry signal of cells infected with the T4SS mutant at a high MOI did not match that of macrophages infected with the complemented strain (see Fig. S4B), which was equivalent to that of wild type (see Fig. S4C). For the mCherry-high population of RAW cells, the mCherry MFI was significantly higher for the macrophages infected with the complemented strain (see Fig. S4B). This suggests that increasing the MOI cannot fully compensate for the intracellular replication defect of the T4SS mutant. Thus, 2-NBDG uptake by all cells highly infected with either the mutant or complemented strain could not be compared, since 2-NBDG uptake is correlated with the level of infection (Fig. 3B). To overcome this difference, we compared cells infected with either the mutant or the complemented strain in two ways. First, we compared glucose uptake among mCherry-low cells. For the mCherry-low cells, the mCherry MFI was significantly higher for the cells infected with the *virB2* mutant than those infected with the complemented strain. However, despite this disparity in bacterial burdens, the macrophages showed equivalent glucose uptake, suggesting that the T4SS promotes the glycolytic switch in infected macrophages (Fig. 6A). Next, for the more highly infected cells, we again binned the data across the range of mCherry signal. Consistent with our previous observation, as bacterial burden increased, the 2-NBDG signal increased. For all bins, the glucose import rate of macrophages infected with the complemented strain was consistently higher than that of macrophages infected with the T4SS mutant (Fig. 6B). Together, these data demonstrate that the T4SS contributes to the glycolytic switch of infected macrophages.

10.1128/mbio.03068-22.4 FIG S4 Comparison of mCherry B. abortus strains. (A) mCherry fluorescence was measured from the mChe-expressing WT, *virB2* mutant, and complemented *virB2* strains, and the fluorescence signal was normalized to viable organisms as measured by CFUs. No statistical difference (ns), one-way ANOVA. (B and C) RAW 264.7 cells were infected with indicated strains at an MOI of 100 for the T4SS-sufficient strains and an MOI of 2,000 for the T4SS-deficient strains for 48 h. (B, left) Representative fluorescence-activated cell sorting (FACS) plots showing the identification of mCherry-negative, mCherry-low, and mCherry-high populations of RAW 264.7 cells infected as indicated. RAW 264.7 cells infected with the nonfluorescent complemented strain is shown as an mCherry-negative control. (Right) mCherry MFI for the mCherry-high population of RAW 264.7 cells infected with the indicated B. abortus strains. **, *P ≤ *0.01 (Student two-tailed *t* test). (C) Representative FACS plots showing the identification of mCherry-negative, mCherry-low, and mCherry-high populations of RAW 264.7 cells infected with WT or the complemented *virB2* strain. RAW 264.7 cells infected with the nonfluorescent WT strain is shown as an mCherry-negative control. Download FIG S4, TIF file, 1.1 MB. Copyright © 2022 English et al. 2022 English et al. https://creativecommons.org/licenses/by/4.0/ This content is distributed under the terms of the Creative Commons Attribution 4.0 International license.

### Impaired induction of glycolysis does not necessarily impact intracellular replication of *B. abortus*.

We and others have shown that IRE1α contributes to the intracellular replication of Brucella (23, 25, 26, 31–33) (see also Fig. S1). Intriguingly, it has also been shown that glycolysis in the infected macrophage and lactate catabolism by Brucella also support intracellular replication (2). We wondered if IRE1α was promoting intracellular replication by increasing glycolysis. B. abortus showed no replication defect in XBP1 or TLR4 KO macrophages (see Fig. S5A and B), despite those cells’ reduced glycolytic induction. Thus, reducing the glycolytic rate of infected cells does not necessarily impair the intracellular growth of B. abortus, suggesting that IRE1α promotes the intracellular replication of B. abortus independently of its role in the glycolytic switch.

10.1128/mbio.03068-22.5 FIG S5 Reduced glycolytic induction does not impair B. abortus intracellular growth. Intracellular replication was enumerated by determining the CFU in RAW 264.7 cells (A) or BMDMs (B) of the indicated genotypes. Data points are means of triplicate wells ± the SD. Download FIG S5, TIF file, 0.1 MB. Copyright © 2022 English et al. 2022 English et al. https://creativecommons.org/licenses/by/4.0/ This content is distributed under the terms of the Creative Commons Attribution 4.0 International license.

### Activation of the IRE1α-XBP1s pathway is not sufficient to increase glycolysis.

**FIG 7:**
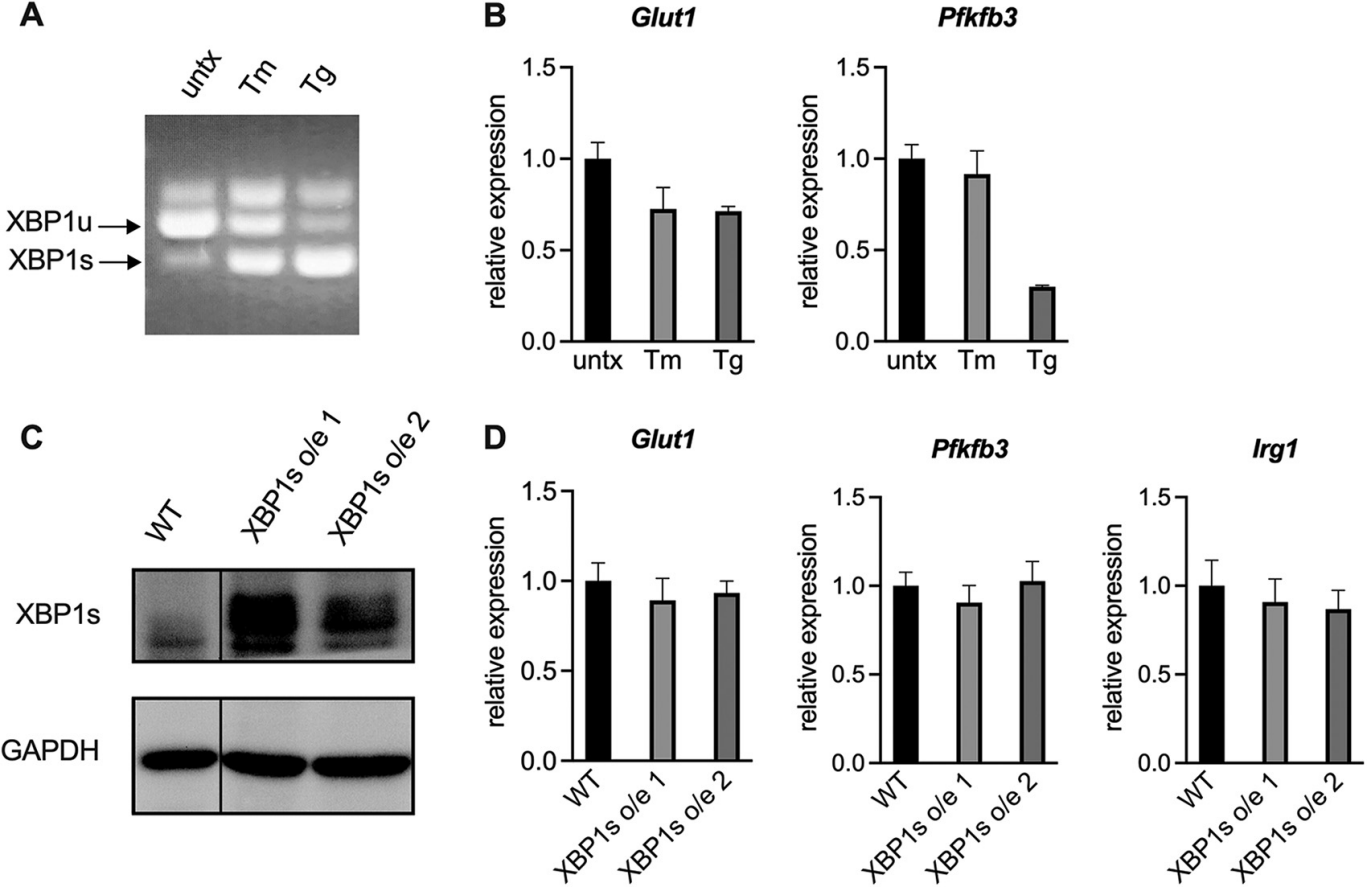
Activation of the IRE1α-XBP1 signaling axis is not sufficient to increase glycolysis in macrophages. (A and B) RAW 264.7 cells were treated with 200 ng/mL tunicamycin (Tm) or 50 nM thapsigargin (Tg) for 24 h. XBP1 splicing was assessed by nonquantitative RT-PCR (A), and expression of the indicated genes normalized to untreated controls was assessed by RT-qPCR (B). (C and D) Two independent XBP1s overexpression (o/e) RAW 264.7 cell lines were generated. XBP1s protein levels were assessed by Western blotting (C), and expression of the indicated genes normalized to wild-type RAW 264.7 was assessed by RT-qPCR (D). The data are means of triplicate wells ± the SD.

Having established that the IRE1α-XBP1 signaling axis promotes glycolysis in CAMs, we then investigated whether activation of this pathway was sufficient to cause increased glycolysis. We treated RAW 264.7 cells with the chemical ER stress inducers tunicamycin and thapsigargin, which led to robust *XBP1* splicing (Fig. 7A). However, neither of these treatments led to an upregulation of glycolytic genes (Fig. 7B), suggesting that IRE1α activation is not sufficient to increase glycolysis.

Tunicamycin and thapsigargin are potent ER stress inducers that activate all three branches of the UPR. To look more specifically at the IRE1α-XBP1 signaling axis, we overexpressed XBP1s in two independently generated RAW 264.7 cell lines (Fig. 7C). As we observed with chemical IRE1α activation, overexpression of XBP1s was not sufficient to upregulate glycolytic genes or the CAM marker *Irg1* (Fig. 7D). Together, these data demonstrate that activation of the IRE1α-XBP1s signaling pathway is not sufficient to increase glycolysis in macrophages.

## DISCUSSION

A cell must utilize the right metabolic pathways to optimally perform its effector functions, and the ability to modulate metabolic processes is essential for cells that must respond to different stimuli, especially immune cells. Here, we show that the ER stress sensor IRE1α and its downstream regulator XBP1s contribute to metabolic reprogramming of macrophages by promoting glycolysis in response to inflammatory stimuli. This occurs when IRE1α is activated in a TLR4-dependent manner, such as with LPS, or a TLR4-independent manner, such as with B. abortus (see Fig. S6). Although TLR4 is not required for IRE1α activation during B. abortus infection, TLR4-deficient macrophages show a reduction in glycolytic flux during infection, suggesting that TLR4 can support glycolysis via IRE1α-dependent and IRE1α-independent mechanisms. TLR4 signaling has been shown to lead to the accumulation of HIF-1α, a key transcriptional regulator of CAMs (50), and the IRE1α-XBP1 signaling axis enhances HIF-1α transcriptional activity without affecting HIF-1α protein levels in cancer cells (37).

10.1128/mbio.03068-22.6 FIG S6 Model of how IREα-XBP1 supports glycolysis in response to inflammatory stimuli. LPS from *S.* Typhimurium signals through TLR4 to activate IRE1α, leading to the production of the transcription factor XBP1s, which supports glycolytic reprogramming. B. abortus uses its T4SS to activate the IRE1α-XBP1s signaling axis, which promotes an increase in glycolysis in infected macrophages. TLR4 also supports glycolysis but is not required for IRE1α activation during B. abortus infection. Dashed lines represent putative IRE1α-independent pathways of TLR4-mediated glycolytic reprogramming, and dotted lines represent putative XBP1s-independent pathways of IRE1α-mediated glycolytic reprogramming. Download FIG S6, TIF file, 0.1 MB. Copyright © 2022 English et al. 2022 English et al. https://creativecommons.org/licenses/by/4.0/ This content is distributed under the terms of the Creative Commons Attribution 4.0 International license.

In this study, we chose to focus on the IRE1α-XBP1 signaling axis; however, because IRE1α activation has effects other than XBP1 splicing, we cannot rule out a role for these other IRE1α functions in metabolic reprogramming. Indeed, while IRE1α-deficient cells showed no increase in lactate production after LPS stimulation or Brucella infection, the XBP1-deficient cells produced an intermediate level of lactate, suggesting that IRE1α may also promote glycolysis via XBP1-independent mechanisms (Fig. 2A and C). For example, IRE1α phosphorylation leads to JNK activation (8), and JNK signaling promotes the Warburg effect in cancer cells (51). In addition to splicing the *XBP1* transcript, activated IRE1α also degrades specific RNA species in a process called regulated IRE1α-dependent decay, or RIDD. Intriguingly, RIDD, which contributes to the intracellular survival of Brucella (28), influences the metabolism of cancer cells (52) and thus may also be affecting CAM metabolism.

While our results demonstrate that the IRE1α-XBP1 signaling axis supports glycolysis in CAMs, it is unclear whether this pathway is also involved in other metabolic changes during macrophage activation. Brucella infection leads to the production of mitochondrial reactive oxygen species (ROS) (53, 54), mitochondrial fragmentation (55), and decreased mitochondrial metabolism (2), while IRE1α activation leads to increased mitochondrial ROS during infection with an attenuated B. abortus strain (53) or multidrug-resistant Staphylococcus aureus (29). However, during B. abortus infection, ROS production and subsequent IL-1β production are XBP1-independent (53), but we show here that XBP1 contributes to glycolysis. On the other hand, XBP1 inhibits mitochondrial function in tumor-associated T cells (15). Future studies will investigate how the IRE1α-XBP1 signaling axis affects mitochondrial function during CAM polarization.

One interesting aspect of this study is the observation that reduced glycolytic induction in macrophages is not sufficient to alter B. abortus intracellular replication. While IRE1α, XBP1, and TLR4 KO macrophages all showed reduced glycolysis during infection, only the IRE1α-deficient macrophages showed a reduced bacterial burden, suggesting that IRE1α supports B. abortus replication independently of enhanced glycolysis. *Irg1* has been implicated in the control of Brucella *in vivo* (56), but we did not observe enhanced replication in macrophages with reduced *Irg1* expression. It has also been reported that inhibition of host cell glycolysis and lactate production with potent small molecule inhibitors impairs Brucella replication (2). However, it is worth noting that IREα and XBP1 KO macrophages are still upregulating glycolytic genes (Fig. 1B, D, F and H; Fig. 2B and D), glucose import (Fig. 3C and D), and glycolytic flux (Fig. 4) under inflammatory stimuli, just to a lesser extent than WT cells. Lactate utilization is required for robust intracellular replication during *in vitro* infection (18). However, during chronic infection *in vivo*, B. abortus favors alternatively activated macrophages (AAM), which have markedly different metabolism compared to CAMs, due to increased glucose availability (4). We believe the ability *of* B. abortus to replicate in cells with different metabolic states contributes to its success as a pathogen. Indeed, different metabolic states of the host cell contribute to the replication of other intracellular pathogens, including Chlamydia trachomatis (57), Salmonella enterica (58), and Legionella pneumophila (59).

It is clear the ER plays a central role in both sensing and directing different metabolic processes and that IRE1α activation can have profound effects on cellular metabolism (60). However, these effects are very context dependent. In NK cells, IRE1α-XBP1 signaling during viral infection drives oxidative phosphorylation mediated by c-Myc (16), while XBP1s inhibits mitochondrial function in tumor-infiltrating T cells (15). In breast cancer cells, XBP1s cooperates with HIF-1α to directly regulate many glycolytic genes, including the glucose importer *Glut1* (37). In obese mice, the IRE1α-XBP1 axis represses AAM polarization (18), and in nonobese mice, it contributes to the mixed phenotype of tumor-associated macrophages, regulating the expression of both CAM and AAM markers (19). By demonstrating that IRE1α-XBP1 signaling is required for robust glycolytic induction in macrophages in response to different inflammatory stimuli, we have provided another context in which this critical signaling pathway plays an important role in immunometabolism.

## MATERIALS AND METHODS

### Bacterial strains and culture conditions.

Bacterial strains in this study are the virulent wild-type B. abortus 2308; its isogenic mCherry+ strain MX2 (4); the T4SS-deficient *virB2* mutant ADH3 (48); its isogenic mCherry+ BCE4; the *virB2* complemented strain ADH8 (48); and its isogenic mCherry+ BCE5. MX2, BCE4, and BCE5 each have an insertion of the pKSoriT-*bla*-*kan*-P*sojA*-*mCherry* plasmid (61). BCE4 and BCE5 were generated via conjugation with S17 Escherichia coli bearing the mCherry plasmid; clones that were kanamycin resistant and fluorescent were selected, and the insertion site was validated by multiplex PCR (the primers are listed in Table S1 in the supplemental material).

10.1128/mbio.03068-22.7 TABLE S1 Oligonucleotides used in this study. Download Table S1, DOCX file, 0.02 MB. Copyright © 2022 English et al. 2022 English et al. https://creativecommons.org/licenses/by/4.0/ This content is distributed under the terms of the Creative Commons Attribution 4.0 International license.

All B. abortus strains were cultured on blood agar plates (UC Davis Veterinary Medicine Biological Media Services) for 3 days at 37°C with 5% CO_2_. B. abortus was then cultured overnight at 37°C with aeration in tryptic soy broth (TSB; BD Difco), then subcultured in acidic EGY (pH 5.5) for 4 h at 37°C with aeration prior to macrophage infections. To confirm equivalent fluorescent signals between MX2, BCE4, and BCE5, each strain was grown in triplicate overnight cultures in TSB, then mCherry fluorescence was measured on a GloMax Explorer Microplate Reader (Promega) and viable cells enumerated by CFU counting after plating on tryptic soy agar (BD Difco) plates and incubation at 37°C with 5% CO_2_ for 3 days. All work with B. abortus was performed at biosafety level 3 and was approved by the Institutional Biosafety Committee at the University of California—Davis.

### Mammalian cell culture.

RAW 264.7 murine macrophage-like cells (TIB-71; ATCC) and their derivatives were cultured in RPMI 1640 media (Gibco) supplemented with 10% heat-inactivated fetal bovine serum (FBS) at 37°C and 5% CO_2_. Bone marrow-derived macrophages (BMDMs) were generated as previously described (4). Briefly, bone marrow cells from femurs and tibiae from 6- to 8-week-old female C57BL/6J (Jackson Laboratory, stock 000664), TLR4 KO (Jackson Laboratory, stock 029015), LysM-Cre^+^ *Ern1^fl/fL^*, and LysM-Cre^−^ *Ern1^fl/fL^* (35) mice were isolated and maintained in RPMI 1640 supplemented with 10% FBS (Gibco), 30% L929 cell supernatant, and GlutaMAX (Gibco) at 37°C and 5% CO_2_ for 7 days before use in *in vitro* assays. The same medium was used for all subsequent BMDM experiments. Lipopolysaccharides from Salmonella enterica serotype Typhimurium (Sigma) and tunicamycin (Sigma) were reconstituted in d-PBS (Gibco). Thapsigargin (Sigma) and 4μ8c (Sigma) were reconstituted in dimethyl sulfoxide. 2-NBDG [(2-*N*-(7-nitrobenz-2-oxa-1,3-diazol-4-yl)amino)-2-deoxyglucose] (Thermo) was reconstituted in 100% ethanol.

### Generation of XBP1 knockout and overexpression cell lines.

To generate XBP1 knockout (KO) cells, complementary oligonucleotides (see Table S1 in the supplemental material) forming the nontargeting control (NTC) sgRNA (5′-TCCTGCGCGATGACCGTCGG-3′) and the XBP1-targeting sgRNA (5′-CGGCCTTGTGGTTGAGAACC-3′) were phosphorylated, annealed, and ligated into BsmBI-digested pXPR_001 (62), resulting in pBCE44 and pBCE41, respectively. To generate lentiviral particles, pBCE44 or pBCE41 were cotransfected with psPAX2 (Addgene, plasmid 12260) and pMD2.G (Addgene, plasmid 12259) using Xfect (TaKaRa Bio) into HEK-293T cells (CRL-2316, ATCC) grown in Dulbecco modified Eagle medium (DMEM; Gibco) with 10% FBS (Gibco). Lentiviral particles were concentrated from clarified supernatants by using a Lenti-X Concentrator (TaKaRa Bio). RAW 264.7 cells were transduced with the lentiviral particles and then selected with 4 μg/mL puromycin (Gibco). After 5 days of selection, single cells were plated by serial dilution in 96-well plates for clonal selection, and gDNA was extracted from the remaining pool by using a DNeasy kit (Qiagen). The *XBP1* locus was amplified by PCR and sequenced by Sanger sequencing using the primers listed in Table S1; the cutting efficiency was estimated by TIDE analysis (63). After confirming efficient disruption, gDNA was prepared from clonal lines using QuickExtract (Lucigen), and the *XBP1* locus was amplified by PCR and sequenced by Sanger sequencing. Putative knockouts were further validated by Western blot analysis and by measuring expression of *ERdJ4*, an XBP1s-specific target, after thapsigargin treatment.

To generate XBP1 overexpression (o/e) lines, *XBP1s* was amplified from cDNA generated from RAW 264.7 cells treated with thapsigargin and cloned into a modified pENTR1A (64; Addgene, plasmid 17398) using Gibson Assembly Master Mix (New England BioLabs). After sequence validation by Sanger sequencing, *XBP1s* was cloned into the mammalian expression vector pLENTI CMV Puro Dest (Addgene, plasmid 17452) using LR Clonase II (Invitrogen). Lentiviral particles were generated, and RAW 264.7 cells were transduced as described above such that two separate XBP1s overexpression lines were generated. XBP1s overexpression was confirmed by Western blot analysis.

### Macrophage infections.

For most infections, 1 day prior to infection, RAW 264.7 cells were seeded in 24-well plates at 5 × 10^4^ cells per well, and BMDMs were seeded at 1.5 × 10^5^ cells per well in 24-well tissue culture plates. For flow cytometry experiments, RAW 264.7 cells were seeded at 2 × 10^5^ cells per well in 6-well tissue culture plates. For infection of RAW 264.7 cells, B. abortus was opsonized for 30 min at room temperature with 20% antiserum in PBS++ prepared from male C57BL/6J mice infected with B. abortus 2308 for 2 weeks. For inoculum preparation, the bacteria were washed in d-PBS (Gibco), diluted in the appropriate cell culture media, and added to the macrophages at an MOI of 100 unless otherwise indicated. The tissue culture plates were then centrifuged at 210 × *g* for 5 min to synchronize infection. After a phagocytosis period of 30 min at 37°C in 5% CO_2_, the cells were washed twice with d-PBS and then incubated with 50 μg/mL gentamicin (Gibco) in the appropriate culture media for 30 min at 37°C in 5% CO_2_; the medium was then replaced with gentamicin-free media. To examine intracellular replication by CFU, infected macrophages were lysed in 0.5% Tween 20 at the indicated time points. The lysates were serially diluted in d-PBS and spread on TSA plates, which were then incubated at 37°C and 5% CO_2_ for 3 to 5 days before colony enumeration. For lactate quantification, cell culture supernatants were sterile-filtered through 0.22-μm-pore size filters and stored at −80°C until use. Lactate levels were measured using a Lactate Colorimetric Assay Kit II (BioVision) according to the manufacturer’s protocol.

### 2-NBDG assay.

RAW 264.7 cells of the indicated genotypes were infected as described above at an MOI of 100 for 2308, ADH8, MX2, and BCE5 or at an MOI of 2,000 for ADH3 and BCE4. After 48 h, the cells were washed three times with d-PBS and collected by scraping. Viable cells were counted on a hemacytometer using trypan blue. One million viable cells were incubated with 300 nM 2-NBDG in glucose-free DMEM (Gibco) with 10% FBS for 45 min, stained with Live/Dead Fixable Aqua (Thermo Fisher) in d-PBS for 15 min, and fixed in CytoFix (BD Biosciences) for 30 min. The cells were then run on a CytoFLEX flow cytometer (Beckman Coulter), and data were analyzed using FlowJo (v10.8.0). Nonfluorescent B. abortus strains were used to inform gating strategies. When indicated for the mCherry-high cells, the mCherry signal was binned by equal units within each log across the population (e.g., 1 × 10^6^, 2 × 10^6^, 3 × 10^6^, etc.).

### Seahorse analysis.

Totals of 2 × 10^4^ RAW 264.7 cells or 3 × 10^4^ BMDMs were seeded in Seahorse XF96 cell culture microplates. The next day, the cells were treated with 100 ng/mL LPS for 6 h in the appropriate media. The cells were assessed on a Seahorse XFe96 Analyzer (Agilent) using a Seahorse XF glycolytic rate assay kit according to the manufacturer’s instructions, with Seahorse XF RPMI (pH 7.4) supplemented with 1 mM pyruvate, 2 mM glutamine, and 10 mM glucose. GlycoPER was calculated using the glycolytic rate assay report generator. All reagents were from Agilent.

### RNA isolation and RT-PCR.

For RNA isolation, cells were washed with d-PBS and collected in TRI Reagent (Molecular Research Center). After the addition of chloroform, total RNA was isolated from the aqueous phase using Econo-Spin columns (Epoch Life Science) and subjected to on-column PureLink DNase (Invitrogen) digestion. To generate cDNA, 1 μg of total RNA was reverse transcribed with MultiScribe reverse transcriptase (Applied Biosystems) with random hexamers (Invitrogen) and RNaseOUT (Invitrogen). Real-time PCR was performed using SYBR green (Applied Biosystems) and the primers listed in Table S1 on a ViiA 7 real-time PCR system (Applied Biosystems) with the following cycling parameters: 50°C (2 min), 95°C (10 min), 40 cycles of 95°C (15 s) and 60°C (1 min), followed by dissociation curve analysis. Data were analyzed using QuantiStudio Real-Time PCR software v1.3 (Applied Biosystems) and analyzed using the ΔΔ*C_T_* method. Isoforms of *XBP1* were detected using nonquantitative RT-PCR with the primers listed in Table S1 and Phusion High-Fidelity PCR Master Mix (Thermo Fisher) under the following cycling conditions: 98°C for 30 s, 35 cycles of 98°C (10 s), 65°C (30 s), and 72°C (30 s), followed by 72°C for 10 min. The resulting amplicons were separated and visualized on a 2.5% agarose gel containing SYBR Safe (Invitrogen).

### Protein isolation and Western blots.

For the TLR4 KO BMDMs, proteins were extracted from samples collected in TRI Reagent (Molecular Research Center) according to a modified protocol (65). For validation of the XBP1 KO and overexpression lines, proteins were extracted using radioimmunoprecipitation assay buffer (50 mM Tris, 150 mM NaCl, 0.1% sodium dodecyl sulfate (SDS), 0.5% sodium deoxycholate, and 1% Triton X-100) with Protease Inhibitor Cocktail Set III, Animal-Free (EMD Millipore). Insoluble debris was removed by centrifugation. Protein concentrations were determined using a Pierce MicroBCA protein assay kit (Thermo Fisher). Equivalent amounts of protein were separated by SDS-PAGE and transferred to Immobilon-P polyvinylidene difluoride membrane (Millipore). Membranes were incubated with antibodies per the manufacturer’s suggestions. Blots were developed with Western Lightning Plus ECL (Perkin-Elmer). The following antibodies were used: XBP1s (Cell Signaling Technology D2C1F, catalog no. 12782), IRE1α (Cell Signaling Technology 14C10, catalog no. 3294), GAPDH (Cell Signaling Technology 14C10, catalog no. 2118), and goat anti-rabbit horseradish peroxidase (Jackson ImmunoResearch). Images were processed with Adobe Photoshop, which was utilized on occasion to change the order of lanes in the image to group appropriate samples together.

### Data analysis.

Data were analyzed with Microsoft Excel (Microsoft) and Prism (GraphPad) using the statistical tests indicated in the figure legends. Densitometry was measured with ImageJ (version 1.53 [66]). Data presented here are from a minimum of triplicate measurements from representative experiments.
