# Supplementary figures and images for: The IRE1α-XBP1 Signaling Axis Promotes Glycolytic Reprogramming in Response to Inflammatory Stimuli
Source: mBio. 2022 Dec 8;14(1):e03068-22. doi: 10.1128/mbio.03068-22 (PMC9973330; doi:10.1128/mbio.03068-22)

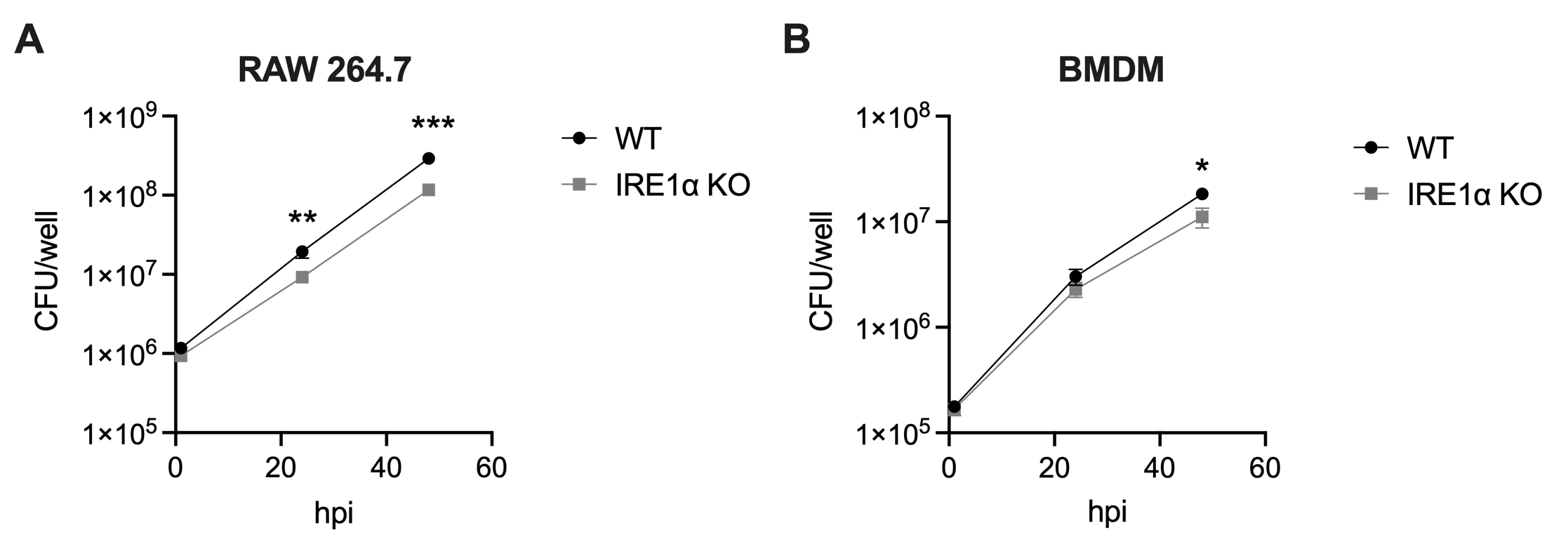

Supplement: FIG S1 [file mbio.03068-22-s0001.tif]

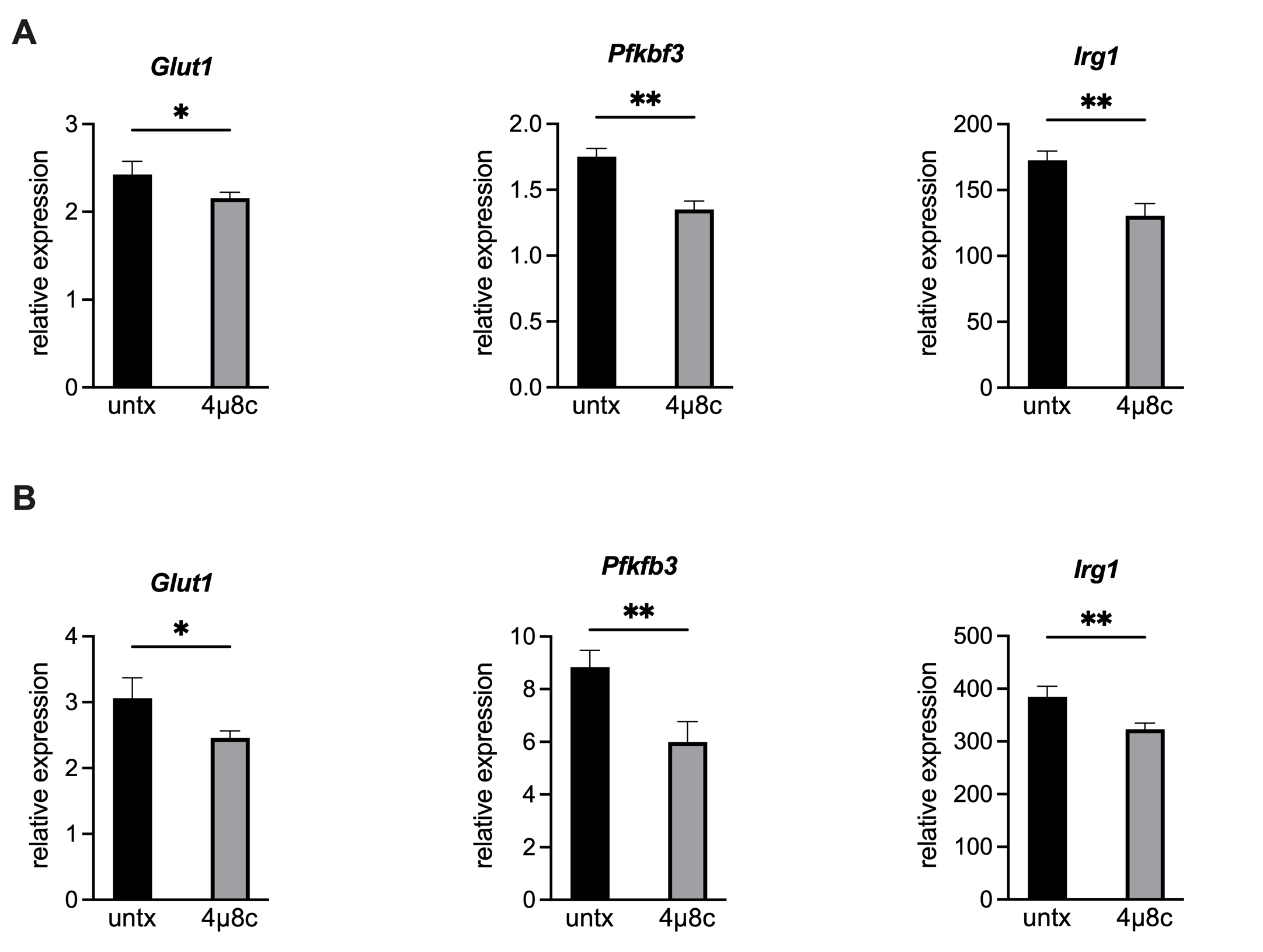

Supplement: FIG S2 [file mbio.03068-22-s0002.tif]

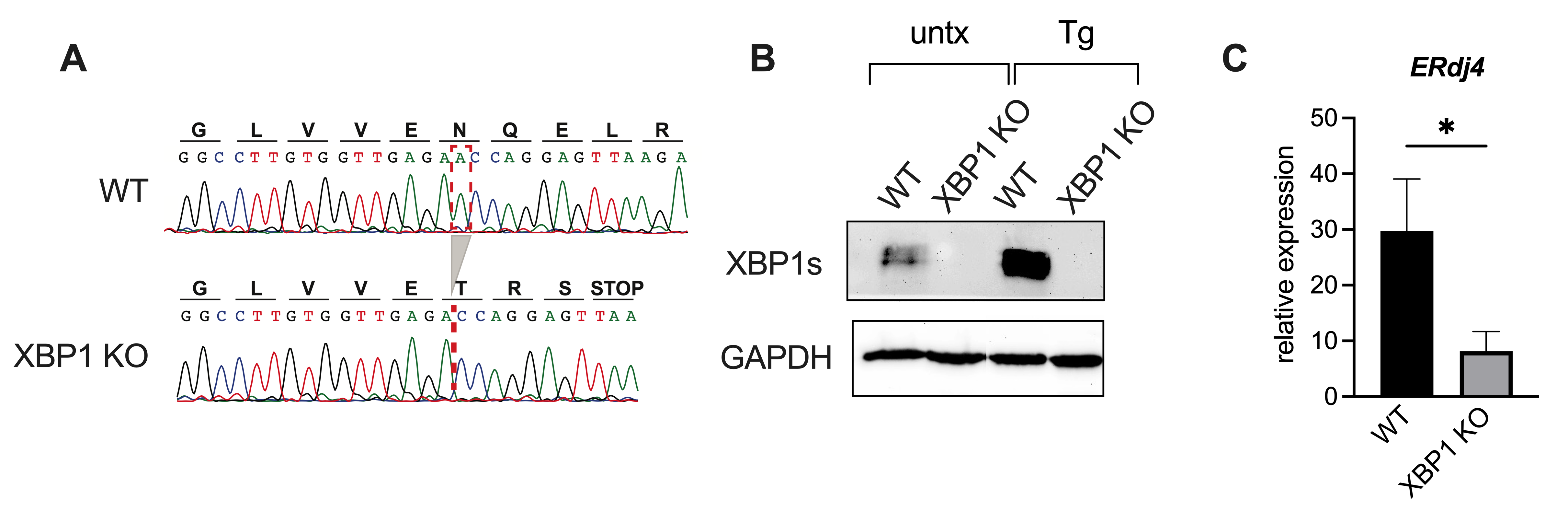

Supplement: FIG S3 [file mbio.03068-22-s0003.tif]

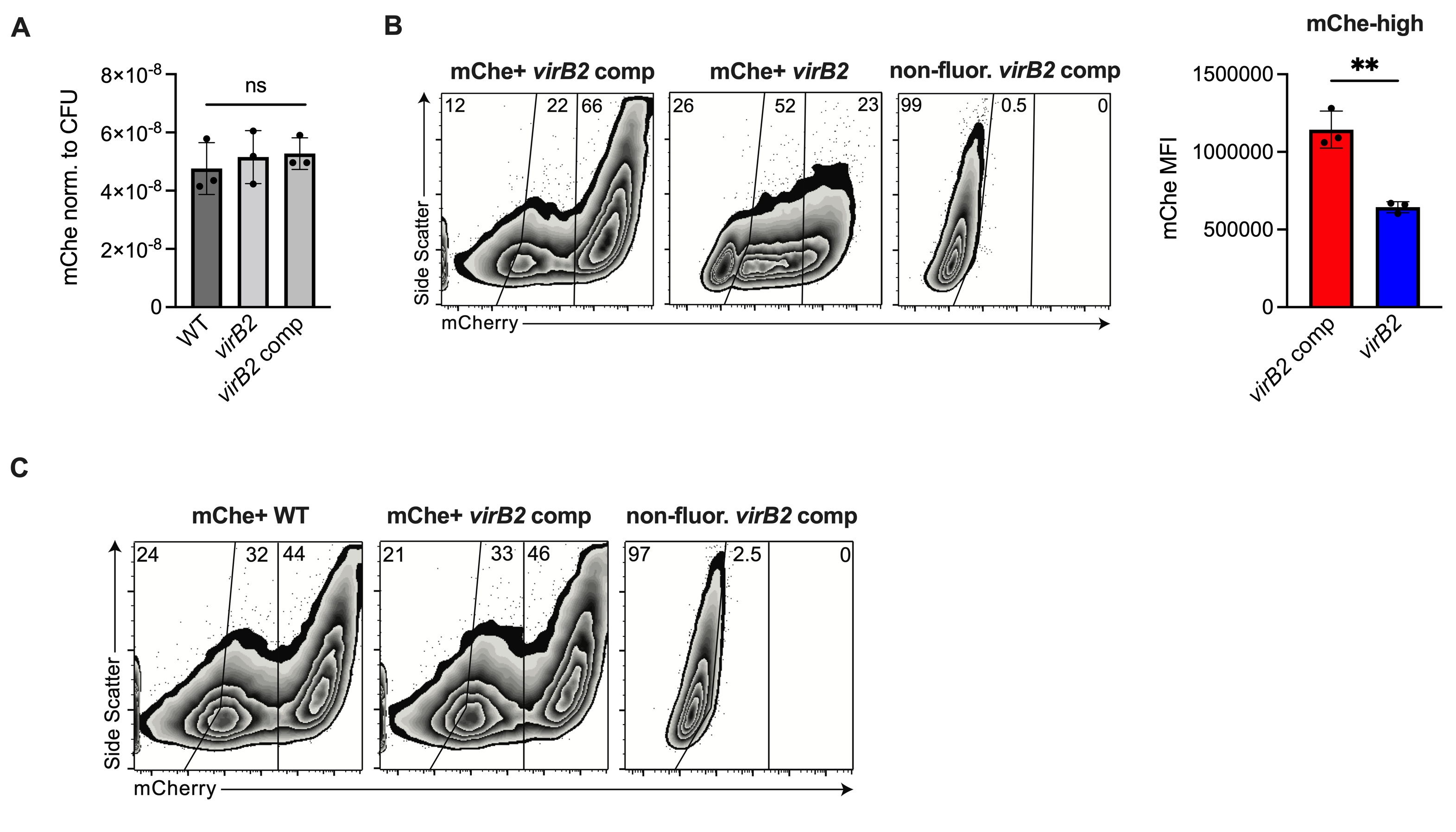

Supplement: FIG S4 [file mbio.03068-22-s0004.tif]

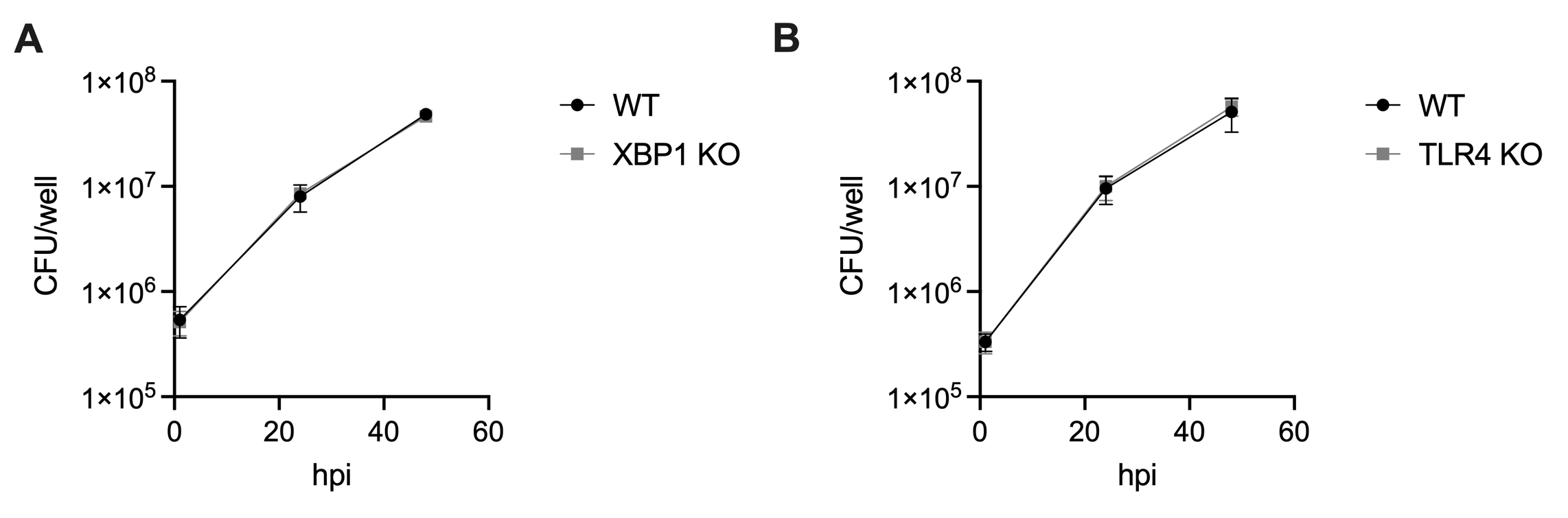

Supplement: FIG S5 [file mbio.03068-22-s0005.tif]

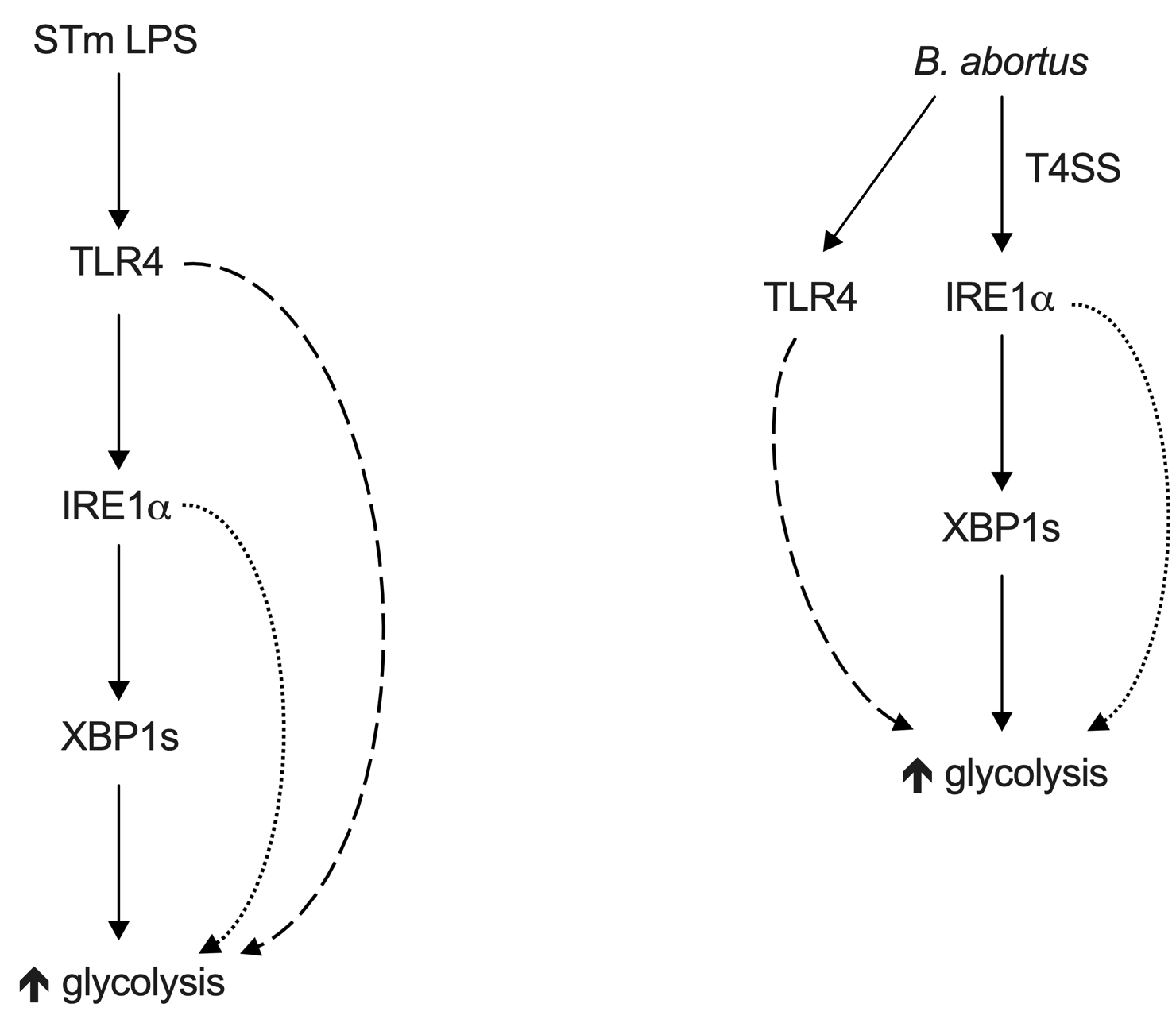

Supplement: FIG S6 [file mbio.03068-22-s0006.tif]
