# Supplementary material for: The IRE1α-XBP1 Signaling Axis Promotes Glycolytic Reprogramming in Response to Inflammatory Stimuli
Source: mBio. 2022 Dec 8;14(1):e03068-22. doi: 10.1128/mbio.03068-22 (PMC9973330; doi:10.1128/mbio.03068-22)
Supplement: TABLE S1 [file mbio.03068-22-s0007.docx]

**Table S1: Oligonucleotides used in this study**

| **Name** | **Sequence (5' - 3')** | **Purpose** | **Source** |
| --- | --- | --- | --- |
| SecE up | GGTCATAATTCCGGCTTCAA | validation of pKSoriT-bla-kan-PsojA-mCherry insertion site | this study |
| SecE down | ATAAGCTGGTCGGCAAGAAA | validation of pKSoriT-bla-kan-PsojA-mCherry insertion site | this study |
| mChe | AAGCGCATGAACTCCTTGAT | validation of pKSoriT-bla-kan-PsojA-mCherry insertion site | this study |
| ActB F | AGAGGGAAATCGTGCGTGAC | RT-qPCR | (1) |
| ActB R | CAATAGTGATGACCTGGCCGT | RT-qPCR | (1) |
| Glut1 F | GCTGTGCTTATGGGCTTCTC | RT-qPCR | (1) |
| Glut1 R | CACATACATGGGCACAAAGC | RT-qPCR | (1) |
| Pfkfb3 F | AGCTGCCCGGACAAAACAT | RT-qPCR | (1) |
| Pfkfb3 R | CTCGGCTTTAGTGCTTCTGGG | RT-qPCR | (1) |
| Irg1 F | GCAACATGATGCTCAAGTCTG | RT-qPCR | (2) |
| Irg1 R | TGCTCCTCCGAATGATACCA | RT-qPCR | (2) |
| IL6 F | GAGGATACCACTCCCAACAGACC | RT-qPCR | this study |
| IL6 R | AAGTGCATCATCGTTGTTCATACA | RT-qPCR | this study |
| XBP1 F | GGCCTTGTGGTTGAGAACCAGGAG | XBP1 splicing | (3) |
| XBP1 R | GAATGCCCAAAAGGATATCAGACTC | XBP1 splicing | (3) |
| mNTC sgRNA F | CACCGTCCTGCGCGATGACCGTCGG | NTC sgRNA | (4) |
| mNTC sgRNA R | AAACCCGACGGTCATCGCGCAGGAC | NTC sgRNA | (4) |
| XBP1 sgRNA F | CACCGCGGCCTTGTGGTTGAGAACC | XBP1 sgRNA | this study |
| XBP1 sgRNA R | AAACGGTTCTCAACCACAAGGCCGC | XBP1 sgRNA | this study |
| XBP1 seq F | GGCTGAAATCTGCGAGTAGTA | XBP1 locus amplification/sequencing | this study |
| XBP1 seq R | AGGAACATCTGCCTGTAATGG | XBP1 locus amplification/sequencing | this study |
| XBP1 Gibson F | CTTTAAAGGAACCAATTCAGTCGACGCCACCATGGTGGTGGTGGCAGCG | XBP1s amplification for Gibson assembly | this study |
| XBP1 Gibson R | GGTCTAGATATCTCGAGTGCGGCCGCTTAGACACTAATCAGCTGGGGG | XBP1s amplification for Gibson assembly | this study |

**TABLE S1 REFERENCES**

1. Xavier MN, Winter MG, Spees AM, den Hartigh AB, Nguyen K, Roux CM, Silva TM, Atluri VL, Kerrinnes T, Keestra AM, Monack DM, Luciw PA, Eigenheer RA, Baumler AJ, Santos RL, Tsolis RM. 2013. PPARgamma-mediated increase in glucose availability sustains chronic Brucella abortus infection in alternatively activated macrophages. Cell Host Microbe 14:159-70.

2. Mills EL, Ryan DG, Prag HA, Dikovskaya D, Menon D, Zaslona Z, Jedrychowski MP, Costa ASH, Higgins M, Hams E, Szpyt J, Runtsch MC, King MS, McGouran JF, Fischer R, Kessler BM, McGettrick AF, Hughes MM, Carroll RG, Booty LM, Knatko EV, Meakin PJ, Ashford MLJ, Modis LK, Brunori G, Sevin DC, Fallon PG, Caldwell ST, Kunji ERS, Chouchani ET, Frezza C, Dinkova-Kostova AT, Hartley RC, Murphy MP, O'Neill LA. 2018. Itaconate is an anti-inflammatory metabolite that activates Nrf2 via alkylation of KEAP1. Nature 556:113-117.

3. English BC, Van Prooyen N, Ord T, Ord T, Sil A. 2017. The transcription factor CHOP, an effector of the integrated stress response, is required for host sensitivity to the fungal intracellular pathogen Histoplasma capsulatum. PLoS Pathog 13:e1006589.

4. Abuaita BH, Schultz TL, O'Riordan MX. 2018. Mitochondria-Derived Vesicles Deliver Antimicrobial Reactive Oxygen Species to Control Phagosome-Localized Staphylococcus aureus. Cell Host Microbe 24:625-636 e5.
